# Perinatal asphyxia and its associated factors in Ethiopia: a systematic review and meta-analysis

**DOI:** 10.1186/s12887-020-02039-3

**Published:** 2020-03-24

**Authors:** Fikadu Waltengus Sendeku, Getnet Gedefaw Azeze, Selamawit Lake Fenta

**Affiliations:** 1 Department of Midwifery, College of Medicine and Health Sciences, Bahir Dar University, Bahir Dar, Ethiopia; 2 Department of Midwifery, College of Health Sciences, Woldia University, Woldia, Ethiopia

**Keywords:** Birth outcomes, Neonatal, Meta-analysis, Morbidity, And mortality, Systemic review

## Abstract

**Background:**

Despite different preventive strategies that have been implemented in different health institutions in the country, neonatal mortality and morbidity are still significantly increasing in Ethiopia. Perinatal asphyxia is the leading cause of neonatal morbidity and mortality worldwide. As a result, this systematic review and meta-analysis aimed to assess the prevalence and associated factors of perinatal asphyxia in Ethiopia.

**Methods:**

Online databases (PubMed, HINARI, EMBASE, Google Scholar and African Journals), other gray and online repository accessed studies were searched using different search engines. Newcastle-Ottawa Quality Assessment Scale (NOS) was used for critical appraisal of studies. The analysis was done using STATA 11 software. The Cochran Q test and I^2^ test statistics were used to test the heterogeneity of studies. The funnel plot and Egger’s test were used to detect publication bias of the studies. The pooled prevalence of perinatal asphyxia and the odds ratio (OR) with a 95% confidence interval was presented using forest plots.

**Result:**

Nine studies were included in this review, with a total of 12,249 live births in Ethiopia. The overall pooled prevalence of perinatal asphyxia in Ethiopia was 24.06% (95 95%CI: 18.11–30.01). Associated factors of perinatal asphyxia included prolonged labor (OR = 2.79, 95% CI: 1.98, 3.93), low birth weight (OR = 6.52, 95% CI: 4.40, 9.65), meconium-stained amniotic fluid (OR = 5.91, 95% CI: 3.95, 8.83) and instrumental delivery (OR = 4.04, 95% CI: 2.48, 6.60) were the determinant factors of perinatal asphyxia in Ethiopia.

**Conclusions:**

The overall pooled prevalence of perinatal asphyxia was remarkably high. Duration of labor, meconium-stained amniotic fluid, instrumental deliveries, and birth weight were the associated factors of perinatal asphyxia in Ethiopia. Therefore, efforts should be made to improve the quality of intrapartum care service to prevent prolonged labor and fetal complications and to identify and make a strict follow up of mothers with meconium-stained amniotic fluid. This finding is important to early recognition and management of its contributing factors, might modify hypoxic-ischemic encephalopathy and may improve the implementation of the standard guideline effectively and consistently.

## Background

Perinatal asphyxia is the failure of neonates to initiate and sustain breathing at birth which causes significant neonatal morbidity and mortality to the extent of the first week of life [1]. Perinatal asphyxia is a condition that occurs when there is an impairment of blood gas exchange that results in hypoxemia and hypercapnia. Moreover, perinatal asphyxia requires adequate and quick resuscitation measures [2, 3]. The combination of hypoxia and ischemia will result in a cascade of biochemical changes in the body leading to brain neuronal death and brain damage during the antepartum or intrapartum period [4].

According to the World Health Organization (WHO), there were nearly four to nine million newborns develop birth asphyxia each year. APGAR score is used to determine the level of perinatal asphyxia, evaluated in the 1st and 5thminutes of life with scores ranging from zero to ten. Four to seven APGAR score in the first minute of life indicates moderate perinatal asphyxia and between zero and three indicates severe asphyxia [5].

According to WHO, perinatal asphyxia is characterized by profound metabolic acidosis, PH < 7.20 on umbilical cord arterial blood sample, the persistence of an APGAR score of 3 at the 5th minute, clinical neurologic sequelae in the immediate neonatal period, evidence of multi-organ system dysfunction (brain damage, heart, lungs, gut, kidneys) in the immediate neonatal period. Pre-pregnancy risk factors for perinatal asphyxia are; maternal age ≥ 35 years, social factors, family history of neurologic disease, infertility treatment, previous neonatal death, chronic illness. Whereas antepartum risk factors include; maternal thyroid disease, severe preeclampsia, multiple gestation, chromosomal/genetic abnormalities, congenital malformations, intrauterine growth restriction, trauma, malpresentation, and antepartum hemorrhage. Besides, chorioamnionitis, meconium-stained amniotic fluid, operative vaginal delivery, general anesthesia, emergency cesarean delivery, placental abruption, cord accident, uterine rupture, maternal cardiac arrest, and fetal exsanguinations were the intrapartum risk factors for perinatal asphyxia [3, 6].

In low and middle-income countries neonatal mortality rate constitutes 42% of under-5 deaths [1, 6, 7]. According to the World Health Organization report, perinatal asphyxia is the third leading cause of under-5 child deaths (11%) following preterm birth (17%) and pneumonia (15%) [2, 6, 8]. In low and middle-income countries, neonatal deaths accounted for 52% of all under-5 child mortality in South Asia, 53% in Latin America and Caribbean, and 34% in Sub-Saharan Africa due to preventable causes including perinatal asphyxia [9].

Aslam HMSS et. al [3] in Ethiopia, perinatal asphyxia is one of the leading causes of neonatal mortality, which accounts for 34% [10]. Therefore, this systematic review and meta-analysis aimed to estimate the pooled prevalence of perinatal asphyxia and to identify the determinant factors in Ethiopia.

## Methods

### Search strategy

International Online databases (Pub Med, HINARI, EMBASE, Google Scholar and African Journals) were used to search articles on perinatal asphyxia. Searching terms were based on adapted PICO questions to search through the aforementioned databases to accesses all the important articles. Different Boolean operators have been established to have comprehensive datasets on perinatal asphyxia. MeSH engines including “perinatal asphyxia” OR “birth asphyxia OR “neonatal asphyxia,” OR “meconium-stained amniotic fluid” OR“ mode of delivery”, OR “preterm birth delivery”, OR “low birth weight” OR “Small for gestational age”, OR “prolonged labor” OR “hypoxic-ischemic encephalopathy” AND related in Ethiopia were searched in the international online electronic database and online university repositories.

### Inclusion and exclusion criteria

Both cross-sectional and case-control studies were incorporated. Studies reported the prevalence and/or associated factors, or determinant factors of perinatal or birth asphyxia or hypoxic-ischemic encephalopathy were included in this study. Only English language literature and research articles were included. Whereas articles without full text and abstract, duplicated studies, anonymous reports, and editorial reports were excluded.

### Data extraction and quality assessment

After collecting findings from all databases, the articles were exported to Microsoft Excel spreadsheet. Two authors (GG & FW) independently extracted the data and reviewed all the screened and included articles. Any disagreement was handled by the third reviewer (SL). After all, a consensus was reached through discussion between authors. Newcastle-Ottawa Quality Assessment Scale (NOS) for cross-sectional and case-control studies is used to assess the methodological quality of a study and to determine the extent to which a study addressed the possibility of bias in its design, conduct, and analysis. All authors independently assessed the articles for inclusion in the review. All of the included articles scored (NOS) seven and more can be considered a “good” study and low risk. However, in this study, all articles were included since they scored more than seven and above of the NOS criteria [11].

### Outcome of measurement

The measurement outcome of this study had two main outcome variables. Perinatal asphyxia or hypoxic-ischemic encephalopathy was the first outcome of the study whereas associated factors of perinatal asphyxia or hypoxic-ischemic encephalopathy was the second outcome of the study. The odds ratio was calculated for the common factors of the reported studies. The outcome of this study was to focus on single studies estimating the prevalence of perinatal asphyxia among live birth neonates.

#### Hypoxic-ischemic encephalopathy/HIE/

Hypoxic-ischemic encephalopathy in a newborn was defined as a 5-min Apgar score of less than 5 and evidence of metabolic acidosis associated with one or more of the following signs of neurologic dysfunction: depression of the level of consciousness, respiratory depression, abnormality of muscle tone, disturbances of cranial nerve function and seizures (in the first week of life) [12].

### Publication bias and heterogeneity

The Cochrane Q test and I^2^ with its corresponding *p*-value were used to assess the heterogeneity of the study. A value of 25, 50, and 75% was used to declare the heterogeneity test as low, medium and high heterogeneity respectively. Funnel plot and Egger regression asymmetry tests were employed to assess the existence of publication bias. As a result, the random effect model of analysis was computed with the evidence of heterogeneity [11].

### Data analysis

The data were entered using Microsoft Excel. This systematic review and meta-analysis were conducted using Stata 11 software. The estimated prevalence of each study was presented using forest plots with a 95% confidence interval (CI). Moreover, subgroup analysis was computed using the study region and the sample size of the studies, with the evidence of heterogeneity.

## Results

### Characteristics of the included studies

234 studies were retrieved at Pub Med, HINARI, EMBASE, Google Scholar and African Journals and other gray and online repository accessed articles regarding the prevalence and risk factors of perinatal asphyxia in Ethiopia. After duplicates were expunged, 113 studies remained.

**Fig. 1:**
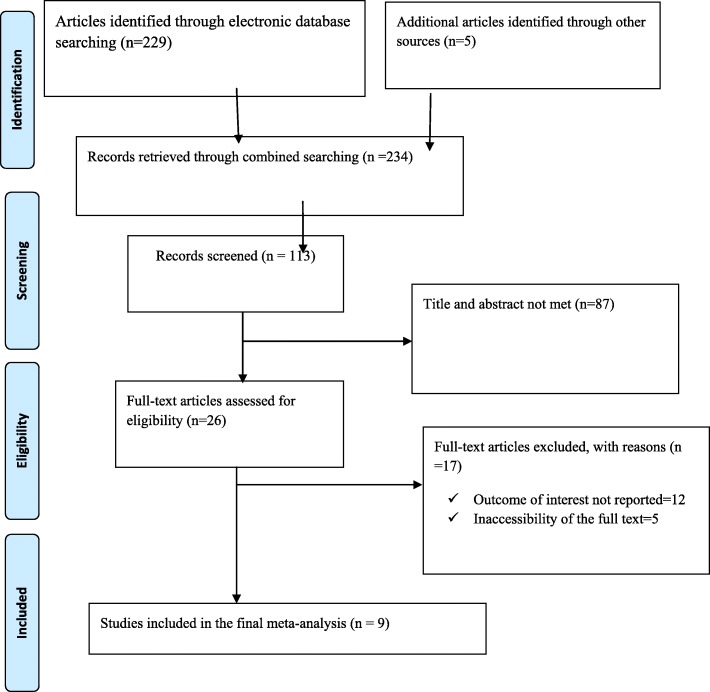
PRISMA Flow chart of study selection for systematic review and meta-analysis of determinant factors of perinatal asphyxia in Ethiopia

Out of the remaining 113 articles, 87 articles were excluded after review of their titles and abstracts. Therefore, 26 full-text articles were accessed and assessed for inclusion criteria, which resulted in the further exclusion of 17 articles primarily due to reason. As a result, 9 studies were met the inclusion criteria to undergo the final systematic review and meta-analysis (Fig. 1). This systematic review and meta-analysis consist of five cross-sectional and four case-control studies. Studies were conducted from different regions of Ethiopia (Amhara, Oromia, SNNPR (South Nation Nationalities people and representatives), Tigray and Dire Dawa. Overall, this review included a total of 12,249 neonates in Ethiopia **(**Table 1).

**Table 1:** Study characteristics included in the systematic review and meta-analysis

No	Authors	Study year	Study design	live births	Region	Gestational age	Study area	P(95% CI)
1	Yohannes K et al	2018	Case-control	380	Amhara	Pre-term = 48 Term =318 Post-term =14	General & referral hospitals, Dessie, Amhara region	–
2	Abebe A et al	2017	Cross-sectional	256	SNNPR	Pre-term = 66 Term =153 Post-term =33	Dilla Universty referral hospital, SNNPR	32.80 (27.12, 38.48)
3	Neil B et al	2014–2017	Cross-sectional	9738	Dire Dawa	Pre-term = 36 Term =210 of the total 246 asphyxiated babies	Dilchora Referral hospital, Dire Dawa	25.00 (24.14, 25.86)
4	Worku N et al	2017	Cross-sectional	154	Amhara	Pre-term = 20 Term &post-term = 134	General hospital Amhara region	29.90 (22.67, 37.13)
5	Lisanu W et al	2017	Case-control	270	Amhara	Pre-term = 27 Term =217 Post-term =26	Gondar referral hospital, Amhara region	–
6	Zelalem J et al	2015	Cross-sectional	371	Oromia	Reported only live birth without GA	General, referral & primary hospitals, Oromia region	12.50 (9.13, 15.87)
7	Gdiom G et al	2018	Cross -sectional	421	Tigray	Pre-term = 25 Term =396	General hospital Tigray region	22.10 (18.14, 26.06)
8	Alemwork D et al	2018	Case-control	386	Amhara	Pre-term = 37 Term =326 Post-term =23	Amhara region hospitals General, referral & primary	–
9	Hagos T et al	2018	Case-control	264	Tigray	Pre-term = 89 Term =175	General, referral & primary hospitals, Tigray region	–

### Epidemiology of perinatal asphyxia in Ethiopia

**Fig. 2:**
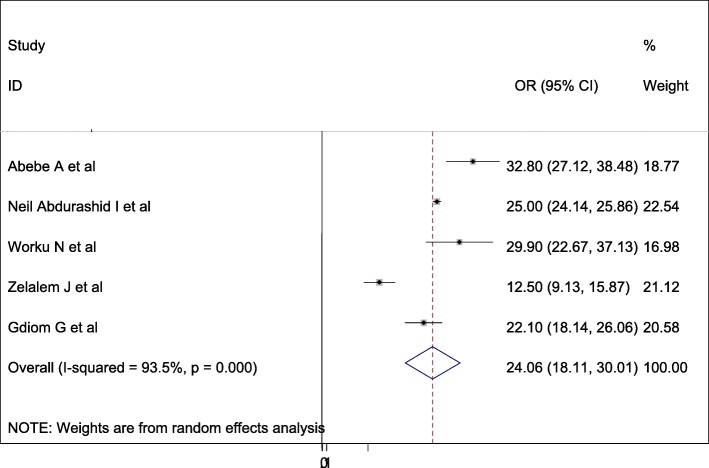
Forest plot displaying the pooled prevalence of perinatal asphyxia in Ethiopia

A wide-ranging prevalence of perinatal asphyxia was observed across different studies included in this review. The overall pooled prevalence of perinatal asphyxia in Ethiopia was 24.06% (95 95%CI: 18.11–30.01, I^2^ = 93.5%, *p* = < 0.001) using a random effect model (Fig. 2).

### Subgroup analysis

**Fig. 3:**
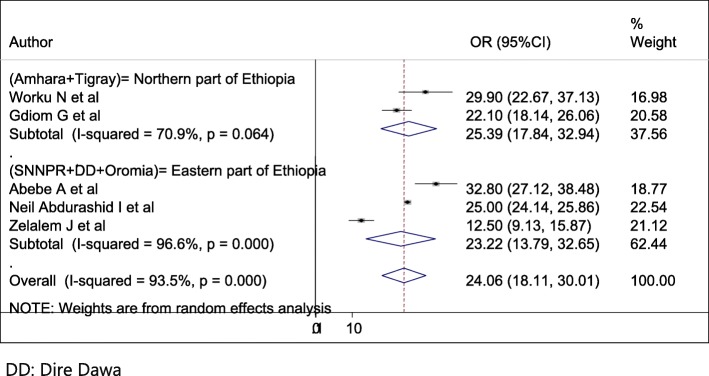
Forest plot of the sub group analysis based on the study area (region)

**Fig. 4:**
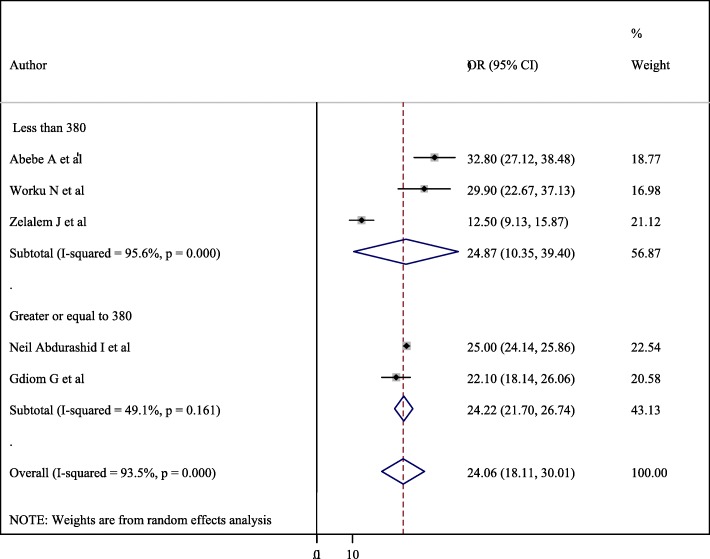
Forest plot of the sub group analysis based on the sample size of the study

Subgroup analysis was conducted based on the study region and sample size. Accordingly, the overall prevalence of perinatal asphyxia by region showed a pooled prevalence of perinatal asphyxia in Amhara & Tigray (Northern part of Ethiopia) was 25.39% (95% CI = 17.84, 32.94, I^2^ = 70%, *p* = 0.046) followed by SNNPR (South Nation Nationalities people representatives) + DD (Dire Dawa) + Oromia (Eastern part of Ethiopia) was 23.22% (95% CI = 13.79, 32.65, I^2^ = 96.6%, *p*- < 0.001) (Fig. 3). Regarding sample size, having less than380 study participants was 24.87(10.35–39.4) with 95%CI, I^2^ = 95.6, *P* < =0.001) (Fig. 4).

### Publication bias

**Fig. 5:**
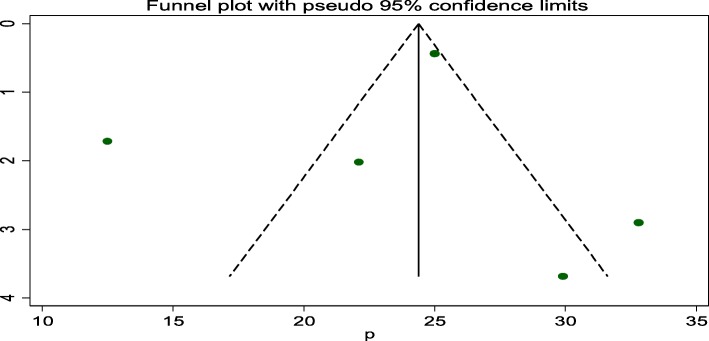
Funnel plot test for publication bias for perinatal asphyxia in Ethiopia

A funnel plot showed that symmetrical distribution and Egger test = 0.763. Hence, there is no publication bias in the studies (Fig. 5).

### Determinants of perinatal asphyxia

#### Relationship between low birth weight and perinatal asphyxia

**Fig. 6:**
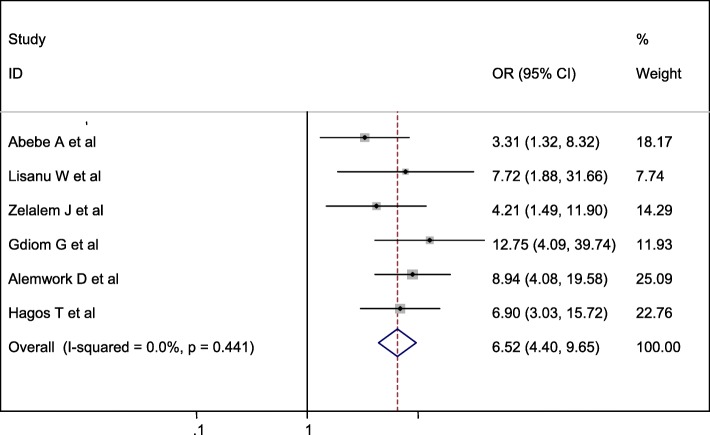
Forest plot displaying the association between low birth weight and perinatal asphyxia in Ethiopia

Six studies were included in this category of meta-analysis [13–18]. Neonates born with low birth weight were 6.52 times (OR: 6.52, 95% CI: 4.40, 9.65) more likely to develop perinatal asphyxia as compared to those born with normal weight. In this meta-analysis, included studies were characterized by no existence of heterogeneity ((I^2^ = 0.0%, *P* = 0.441). Moreover, we used a fixed-effect model analysis due to the absence of heterogeneity **(**Fig. 6).

#### Association between meconium-stained amniotic fluid and perinatal asphyxia

**Fig. 7:**
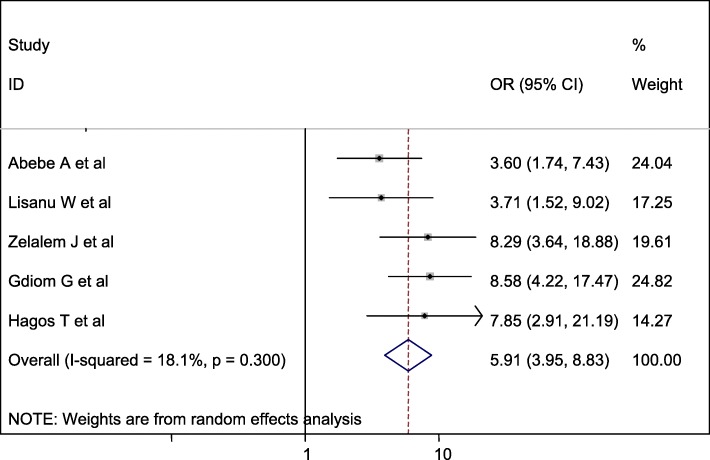
Forest plot displaying the association between MSAF and perinatal asphyxia in Ethiopia

Five studies were included in this category of meta-analysis [13–16, 18]. The likelihood of developing perinatal asphyxia among neonates born with meconium-stained amniotic fluid was 5.91 times (OR = 5.91, 95% CI: 3.95, 8.83) more likely to develop birth asphyxia than the counterparts. In this meta-analysis, included studies were characterized by low heterogeneity (I^2^ = 18.1%, *P* = 0.300), meanwhile, we calculated a random effect meta-analysis (Fig. 7).

#### Association between mode of delivery and perinatal asphyxia

**Fig. 8:**
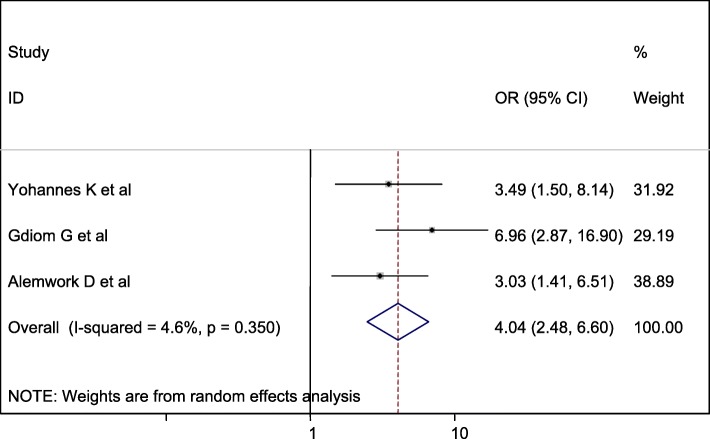
Forest plot displaying the association between assisted instrumental delivery and perinatal asphyxia in Ethiopia

Three studies were included in this category of meta-analysis [16, 17, 19]. The likelihood of developing perinatal asphyxia among neonates born with assisted instrumental delivery was 4.04 times (OR = 4.04, 95% CI: 2.48, 6.60) more likely to develop birth asphyxia than neonates born with spontaneous delivery. In this meta-analysis, included studies were characterized by low heterogeneity (I^2^ = 4.6%, *P* = 0.350). Furthermore, we computed a random effect meta-analysis with the evidence of heterogeneity (Fig. 8).

#### Association between duration of labor and perinatal asphyxia

**Fig. 9:**
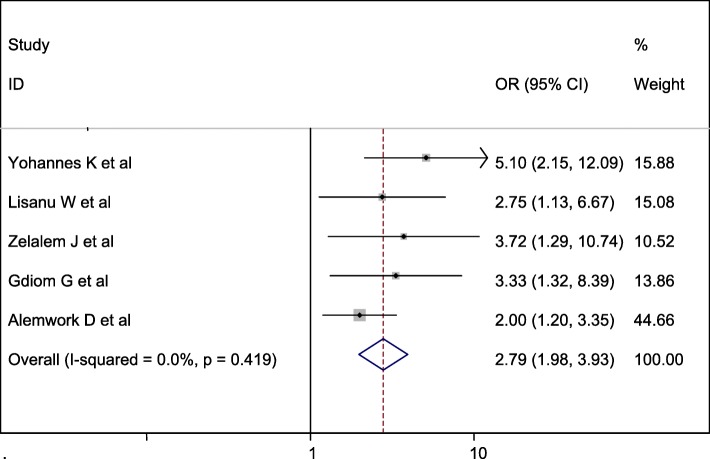
Forest plot displaying the association between prolonged labor and perinatal asphyxia in Ethiopia

Five studies were included in this category of meta-analysis [13, 14, 16, 17, 19]. The odd of developing perinatal asphyxia among neonates born from mothers with prolonged labor was 2.79 times (OR = 2.79, 95% CI: 1.98, 3.93) more compared with their counterparts. In this meta-analysis, included studies were characterized by no possibility of heterogeneity (I^2^ = 0.0%, *P* = 0.419). Therefore, we computed a fixed-effect meta-analysis with the evidence of heterogeneity of the studies (Fig. 9).

## Discussion

According to the results of this meta-analysis, the pooled prevalence of perinatal asphyxia in Ethiopia was estimated to be 24.06% (95% CI: 18.11, 30.01). Practicing standard guidelines during labor and delivery across different hospitals and health institutions could reduce this significant neonatal burden at the national level.

This study finding is in line with the study done in Nigeria reporting that, 30.1% [20] and Tanzania (21.1%) [21] of the neonates were having perinatal asphyxia. The finding of this study is lower than the study conducted in Bangladesh which was estimated 56.9% [22]. The discrepancy might be due to the difference of good utilization of different intrapartum fetal monitoring equipment and tools, having qualified professional and quality of care given services to neonates in Ethiopia.

The finding of this systematic review is higher than the study conducted in Thailand, Malawi and Guinea accounted for 11.7% [23], 6.1% [24] and 2.4% [25] respectively. This discrepancy might be due to socio-cultural, antenatal care service accessibility, and variability in maternal risk factor distribution. Besides, most of the studies included in this meta-analysis were conducted in hospitals and health centers which might increase the prevalence.

Neonates born with low birth weight were 6.52 times (OR: 6.52, 95% CI: 4.40, 9.65) more likely to develop birth asphyxia as compared to neonates born with normal birth weight. Similar study findings were observed in different countries: Uganda [26], Indonesia [27], Pakistan [3], East Java [28] and Thailand [29]**.** This could be explained by because a high proportion of small babies might be pre-term that they might not have enough surfactant which might not prevent suffering from difficulty of breathing and developing difficulty in cardiopulmonary transition and subsequent birth asphyxia.

The likelihood of developing birth asphyxia among neonates born with meconium-stained amniotic fluid was 5.91 times (OR = 5.91, 95% CI: 3.95, 8.83) as likely to have birth asphyxia as those born with clear amniotic. This finding was consistent with the study conducted in India [30, 31] in Iraq [32], and Uganda [26]. The possible reason could be because the intrapartum inhalation of meconium-stained amniotic fluid resulted in chemical pneumonitis with inflammation of the pulmonary tissues, mechanical obstruction of airways, and pulmonary air leak, inducing hypoxia [33].

Newborns delivered with assisted instrumental deliveries 4.04 times (OR = 4.04, 95%CI = 2.48, 6.60) more likely to develop perinatal asphyxia as compared to with neonates delivered spontaneously. This study finding is supported by the study done in India [30] and Iran [34]. The possible explanation for this might be because instrumental delivery causing birth trauma leads to asphyxia. Instrumental deliveries may affect different locations of cranial hemorrhage; subdural, subarachnoid, intra-parenchyma or intra-ventricular due to exerting pressure of a vacuum and forceps extractors can cause brain bleeds on the cranium contributing to intracranial hemorrhage resulting birth asphyxia, bleeding diathesis, infection, and vascular anomalies. As evidenced by a piece of literature, the incidence of traumatic intracranial hemorrhage of six per 1000 live neonates born than spontaneously had born [35].

The odds of birth asphyxia among babies born to mothers with prolonged labor were about 2.79 times (OR = 2.79, 95%CI = 1.98, 3.93) higher than their counterparts. The finding of this study is consistent with the study done in Bangladesh [36], Cameroon [37], Nigeria [20] and Sweden [38]. This could be because if the labor does not progress normally, the laboring mother may experience serious complications, such as dehydration, exhaustion, or rupture of the uterus [39] and if labor becomes prolonged, there is a high chance of the fetus to become distressed.

Despite this, there is a standard guideline for neonatal resuscitation; the death of neonatal mortality is increasing in Ethiopia. In the Ethiopian context with the adoption of the WHO neonatal resuscitation procedures are employed. Initiate cardiac massage at a rate of 120 beats/min, intubation and continuous positive pressure ventilation with 100% of oxygen at a rate of 40 breaths per minute, observe for improvements intermittently (initiation of spontaneous respiration; color, heart rate), administer normal saline and sodium bicarbonate to counter acidosis and shock respectively), and adrenalin through endotracheal tube were the procedures for neonatal resuscitation [40].

This systematic review and meta-analysis had significant heterogeneity which may prone to publication bias. This might be due to the sample size of each study, the nature of the study design, the study setting or region. Despite this review has marked heterogeneity, subgroup analysis was computed using study setting and sample size and showed improvement of marked heterogeneity to a moderate extent, resulting in reducing the having the chance of publication bias.

### Limitation

This systematic review and meta-analysis revealed to estimate the national burden of perinatal asphyxia. However, we were unable to find any neonatal resuscitation measures and effectiveness from the single studies to show the national burden in this review. Including papers only published by the English language and accessing only hospital-based studies was the restraint of the study. It might lack national representativeness because no data were from all regions. Besides, this study included smaller studies (those with larger standard errors) tend to have a chance of having publication bias.

## Conclusion

The overall pooled prevalence of perinatal asphyxia was remarkably higher. Prolonged labor, meconium-stained amniotic fluid, instrumental deliveries, and low birth weight were the determinants factors of perinatal asphyxia in Ethiopia. Therefore, efforts might be made to improve the quality of intrapartum care services to prevent labor, delivery and fetal complications and to identify and make a strict follow up of mothers with meconium-stained amniotic fluid. Addressing and identifying determinants and magnitudes of hypoxic-ischemic encephalopathy may improve the implementation of the WHO standard guideline effectively and consistently.

## Data Availability

All related data has been presented within the manuscript. The dataset supporting the conclusions of this article is available from the authors on request.

## Abbreviations

CI: Confidence Interval
DD: Dire Dawa
MSAF: Meconium stained amniotic fluid
OR: Odds Ratio
PNA: Perinatal asphyxia
WHO: World Health Organization
